# Treatment of Hœmoptysis from Lung Cavities

**Published:** 1878-02

**Authors:** 


### MISCELLANEOUS.

### Treatment of Hœmoptysis from Lung Cavities.

Dr. R. DOUGLAS POWELL, Physician to Brompton Hospital for consumption, makes the following remarks on the treatment of hemorrhage from phthisical cavities. (*Lancet*, Dec. 1, 1877).

The treatment is such as would be dictated by common sense. The most absolute rest in bed is imperative. Beware of the brandy-bottle. The first thing the friends of the patient naturally do when they find him faint from hemorrhage is to give him brandy. But this moment of faintness is just the period at which there is the opportunity for the hemorrhage to become staunched by the formation of a coagulum, and so long as the pulse does not absolutely fail, we should withhold stimulants, and avoid them throughout the treatment of the case. We can scarcely expect drugs to do much in such cases as these. Ergot in full doses and turpentine have been found most useful at this hospital. The momentary application of an ice-bag to the chest or between the shoulders appears sometimes to be useful. When the shock is great, opium will best relieve it. After a day or two, if the exhaustion and anæmia be great, an astringent form of iron is often of great value, as the iron alum or the pernitrate of iron, but the effect of these remedies must be closely watched. In cases in which there is a tendency to recurrence of the hæmoptysis, such patients usually making blood fast, the diet should be carefully restricted, principally to fish and farinaceous food without stimulants.

